# The Herbst Method of Filling Teeth

**Published:** 1885-05

**Authors:** 


### Editorial.

### THE HERBST METHOD OF FILLING TEETH.

Judging by the discussions in some of our dental societies, there is much of misunderstanding and misconception of the principles upon which the rotary method of condensing gold is founded. Before the advent of adhesive foil, fillings of gold were inserted in cavities by the stuffing and wedging process. There was no such thing as condensing the foil into a homogeneous mass, but the laminæ were inserted perpendicular to the axis of the cavity, and so wedged upon each other as to be retained in place. Contour operations were, as a matter of course, impracticable under such manipulation.

When adhesive gold was presented to the profession new methods were introduced, for it is evident that the wedging of cylinders or pellets of this foil would be impossible. The qualities of the gold demanded that a filling should be commenced at the deepest portion of the cavity, and by gradual accretion built to the surface. The mallet was but the natural accompaniment of such gold. All operations that had for their object the making of solid gold fillings, have been inserted upon the principle of the welding of two pieces of homogeneous metal under the impact of a blow, or by the force of a steady pressure. The practice of gradually building up a filling from the bottom, or the use of adhesive foil, has almost entirely superseded the old wedging process. Nearly all dentists of repute to-day fill teeth with foil that is more or less adhesive. The universal practice is to employ the mallet in some way, or to condense by hand pressure, and these methods presuppose a degree of adhesiveness in the foil used.

The Herbst system is quite distinct from either of these methods. The gold must be of the nature of that which was formerly used for the wedging process. It is quite impossible to properly pack this under the mallet, with serrated points, as it "chops" in pieces. The principle is that of burnishing together separate surfaces. Two pieces of gold that could not be made to cohere by hammering with small points, may be thoroughly welded together by the rubbing over them of a smooth instrument, the pieces being held immovably. If this were done by a burnisher held in the hand, it would necessarily be by a reciprocating movement; but a little reflection will enable anyone to perceive that the rotary burnisher accomplishes precisely the same result. And this is the essential principle of the Herbst system.

It is readily conceivable that certain desired objects can be more easily and perfectly accomplished by burnishing than by malleting. If it be desired to unite two pieces of No. 120 gold, lying one upon the other on a flat surface, no one would think of using a serrated plugger and a mallet. He would, holding them immovably, go over them with a smooth burnisher. Again, if it were desired to secure the closest approximation of a pellet or thin plate of gold to the parieties of a cavity, a little reflection will teach any one that it could be more readily and perfectly done by burnishing, than by hammering with a fine point. Theoretically then, in a cavity adapted to its use, the Herbst system must give the most perfect fillings. Practically, however, its application is somewhat limited. It is only in cavities having four good walls that it can readily be used, and these are not the ones requiring the highest skill to successfully fill. Gold having only the comparatively small amount of adhesion that the ordinary soft foil possesses, will not answer for the rotary process, because the surfaces unite too readily, and there is a consequent "balling" of the material; so a special kind of foil is requisite, and it is a fact that gold that will not condense with the mallet, under the rotary burnisher becomes a coherent mass. But in using it the operator must remember that he is employing a manipulation that is not at all adapted to the ordinary gold, and that the process is quite distinct, and therefore requires that he drop all his usual methods.

The advantages of the Herbst system are, that gold can be condensed much more rapidly, and with greater ease to both patient and operator. The relief to the former, especially, is most marked. Its objections are, that it is practically adapted to but a limited range of operations But everyone who aspires to the name of operator, should at least make himself familiar with the principles and method of manipulation, even though he should not practice it habitually. It is not just to unqualifiedly condemn it until one thoroughly comprehends it, and has acquired some familiarity with its method of manipulation.

